# Lens-related ocular emergencies (LROE) in dogs: treatment and visual outcome after late presentation of 90 eyes

**DOI:** 10.1186/s13620-023-00240-1

**Published:** 2023-07-18

**Authors:** Khaled M. Ali, Ayman A. Mostafa

**Affiliations:** Department of Small Animal Surgery and Ophthalmology, Faculty of Veterinary Medicine, Cairo University, PO Box 12211, Giza, Egypt

**Keywords:** Dogs, Glaucoma, Lens-related ocular emergencies, Luxation, Uveitis, Vision

## Abstract

**Background:**

Lens-related emergencies need immediate medical intervention to reduce complications, minimize pain, and improve the chances of retaining vision. The present study aimed to demonstrate the common lens-related ocular emergencies in dogs and evaluate the short-term outcomes after the treatment of these cases. Sixty dogs (90 eyes) of different breeds were presented with unilateral (30 eyes, OD = 18, OS = 12) and bilateral (60 eyes) ocular abnormalities related to crystalline lens injury. Clinical, ultrasonographic, and laboratory examinations were achieved. Different treatment protocols were conducted after a complete ophthalmic examination and the associated clinical outcomes were evaluated.

**Results:**

Mean (± SD) age of dogs at initial evaluation was 3.65 (± 2.4) years (range, 1˗12 years). Lens luxation and subluxation were diagnosed in 45 eyes (25 with anterior lens luxation, 15 with subluxation, and 5 with posterior lens luxation). Lens-induced anterior uveitis without ocular hypertension (*n* = 25 eyes), lens-induced uveitis with secondary glaucoma (uveitic glaucoma) (*n* = 15 eyes), and lens capsule disruption (*n* = 5 eyes) were also diagnosed. The vision was lost in all 5 eyes with posterior lens luxation and secondary glaucoma (100%), 18/25 eyes with anterior lens luxation (72%), and 5/15 eyes with lens subluxation (33.3%). Vision impairment was also identified in 10/25 eyes (40%) with unresponsive lens-induced anterior uveitis and in 5/5 eyes (100%) with traumatic rupture of the anterior lens capsule.

**Conclusion:**

Crystalline lens pathology can cause a wide variety of ocular emergencies that may result in blindness. Early diagnosis and appropriate treatment are crucial for handling lens-related emergencies in dogs.

## Background

The crystalline lens is a smoothly margined, biconvex transparent structure enclosed in its capsule, located immediately behind the iris and in front of the vitreous body [1]. The lens is encircled by ciliary processes and held in place by zonular fibers equatorially, the iris anteriorly, and the anterior vitreous face posteriorly [1]. The lens has a refractive feature that allows for a clear focus of images on the retina. Lens-related emergencies need immediate medical intervention in an attempt to reduce complications, minimize pain and improve the chances of retaining vision [2]. The common lens-related ocular emergencies in dogs and cats include lens capsule disruption [3, 4], lens instability or luxation [5], septic implantation syndrome [6], lens-induced uveitis [7], and secondary glaucoma [8, 9]. Traumatic corneal laceration with associated lens capsule disruption is another common ocular emergency in small animal ophthalmology practice.

Immediate therapy, with the possibility of surgical lens removal, has been deemed crucial to avoid vision-threatening complications [10]. Phacoclastic uveitis, pupillary occlusion, and secondary glaucoma are common complications that often lead to vision loss [10]. In two retrospective studies performed on dogs and cats affected with phacoclastic uveitis, pupillary occlusion, and secondary glaucoma, patients that experienced early lens removal responded favorably [3, 11]. To avoid possible vision impairment, prophylactic lens extraction has previously been recommended if there is a 1.5 mm or greater lens capsule tear or when the lens cortex is significantly damaged [3]. Juvenile cataract develops rapidly in dogs as a consequence of spontaneous cataract resorption [12]. The protein from the lens enters the aqueous to be exposed to the uvea immune system causing lens-induced uveitis [13]. Lens-induced uveitis can therefore be clinically evident or subclinical in cataractous patients [8]. The presence of lipid flare in diabetic cataractous patients as a result of elevated triglycerides or cholesterol, or both, may be the first indicator of inflammation [8].

Lens luxation can be primary or secondary. Primary lens luxation occurs in most Terrier breeds as well as the Shar-pei, Border collie, and German shepherd [5]. Anterior and posterior lens luxation have a more serious influence on vision compared to lens subluxation, with hyperopia, retinal detachment, chronic uveitis, and corneal oedema being the most related complications [14]. The consequences of anterior lens luxation include physical obstruction of aqueous flow, pupillary block glaucoma, and compression of the iridocorneal angle and ciliary cleft by the basal iris [15]. If the lens is in the anterior chamber, it can be removed by intracapsular lens extraction or by phacoemulsification, based on the surgeon’s preference [16]. In posterior lens luxation, the lens appears embedded in the vitreous, non-motile, and in contact with the retina, thereby increasing incidence of retinal damage. However, the chances of developing glaucoma and uveitis remain lower in the posterior compared to anterior lens luxation [17]. Therefore, the objectives of the present study were to demonstrate lens-related ocular emergencies in dogs and to evaluate the short-term clinical and visual outcomes after the treatment of these long-standing cases.

## Methods

### Animals and ophthalmic examination

The study was carried out on 60 (90 eyes) client-owned dogs with lens-related ocular emergencies. These dogs were admitted to the Ophthalmology Service at the Department of Small Animal Surgery, Faculty of Veterinary Medicine, Cairo University from November 2018 to September 2020. The study protocol was approved by the Scientific Committee of Small Animal Surgery at Cairo University before the investigation. No ethical approval was required, as this study investigated a series of client-owned clinical cases admitted to our clinic without the involvement of experimental subjects. Informed consent was obtained from the owners of all enrolled dogs before performing the diagnostic and treatment procedures. Data collected from the clients included signalment, history of trauma, duration of clinical signs, and history of previous medications. Before inclusion, all dogs underwent a complete ophthalmic examination including the slit-lamp examination (SL 14 handheld slit lamp, Kowa, Tokyo, Japan), fluorescein staining (Bio-Glo® Fluorescein sodium Strips 1 mg, HUB pharmaceuticals, LLC., USA), indirect ophthalmoscopy (Riester, Germany), and measurement of intraocular pressure (IOP) using Tonopen tonometer (Tonopen XL®, Reichert Technologies, NY, USA). Topical anesthetic solution (Benoxinate hydrochloride; Benox® 0.4% ophthalmic solution, EIPICO Co., Egypt) was instilled two minutes before IOP measurement. The vision status was evaluated via a menace response, pupillary light reflex, and the history of proper vision before the acute onset of the disease in patients with anterior luxation and existing pressure spike. The enrolled dogs were considered eligible if they were diagnosed with lens subluxation or luxation (anterior or posterior), lens-induced uveitis (LIU), lens capsule disruption, or secondary lens-induced glaucoma. Dogs with immature cataracts without evidence of related ocular abnormalities and those with incurable or infected endophthalmitis were excluded from the study. The clinical findings associated with all enrolled cases were evaluated and recorded.

### Ultrasonographic examination and laboratory tests

The ultrasonographic examination was performed on 15 eyes with concurrent corneal oedema using an ocular B-mode scan and a 7.5–10 MHz micro convex probe (EDAN DUS 60 PRO, digital ultrasound, Shenzhen, P.R. China). Serum triglycerides and cholesterol levels were evaluated in 3 dogs with lipid flare.

### Treatment protocols

#### Lens subluxation/luxation with or without secondary glaucoma

Lens luxation and subluxation were diagnosed in 45 eyes (25 with anterior lens luxation, 15 with subluxation, and 5 with posterior lens luxation) and treatment protocols involved medicinal management and surgical intervention.

#### Medicinal management

Medical treatment was applied to the 15 eyes diagnosed with lens subluxation with (11 eyes) and without (4 eyes) secondary glaucoma, and to the 5 eyes with posterior lens luxation and associated glaucoma. The 11 eyes with lens subluxation and secondary glaucoma and 5 eyes with posterior lens luxation and secondary glaucoma were treated with prostaglandin analog latanoprost (Xalatan®; Pfizer) twice daily [14, 18] and pilocarpine 2% (Isoptocarpine®, Alcon, Egypt). The 4 eyes with lens subluxation and no evidence of secondary glaucoma have received only miotic agents to keep the lens stable behind the corresponding pupil; pilocarpine 2% (Isoptocarpine®, Alcon, Egypt). All treated dogs were re-evaluated at weekly intervals for 3 successive weeks.

#### Surgical intervention

Eyes with anterior lens luxation were treated by routine intracapsular extraction of the lens through a dorsal corneal incision [16]. Associated emergency treatment to lower the concurrent inflammatory process was performed for 19 out of 25 eyes using systemic dexamethasone sodium phosphate (0.1 mg/kg b.w. i.v., Dexamethasone® 8 mg/2ml, Amriya pharmaceuticals, Egypt). Topical application of tobramycin/dexamethasone combination (Tobradex®, Alcon, Egypt), timolol maleate 0.5% (timolol®, EIPICO, Egypt), and tropicamide1% (mydriacyl®, Alcon, Egypt) was performed every 8–12 h for 14 days until the time of surgery. Dogs were routinely pre-medicated with atropine sulphate (Atropine sulphate®; ADWIA, Egypt) and xylazine hydrochloride 2% (Xylaject®; ADWIA, Egypt) in a dose of 0.04 mg/kg b.w. s.c. and 1 mg/kg b.w. i.v., respectively. General anesthesia was achieved with ketamine hydrochloride 5% (Keiran®, EIMC Pharmaceuticals Co., Egypt) in a dose of 15 mg/kg b.w. i.v. A 0.5 ml of sterile, non-pyrogenic, high molecular weight, non-inflammatory highly purified viscoelastic preparation containing sodium hyaluronate (PROVISC®, 0.55 ml sodium hyaluronate1%, Alcon, Egypt) was injected to fill the anterior chamber. The anterior chamber was then entered through a 45° angled slit made by a corneal knife. Angled incisions, varying between 150° and 170°, were made using left- and right-handed corneal scissors, and the lens was grasped and removed using a lens loop. The anterior chamber was then irrigated using sterile compound sodium lactate (Hartmann’s solution®, Braun medical industries, Malaysia), and the corneal incision was closed using 8 − 0 polyglactin (coated vicryl®; Ethicon, USA) in a simple interrupted pattern. All surgical procedures were done under a binocular surgical microscope (12.5x, 66 VISION TECH CO., LTD. China). A systemic course of a 3rd generation of cephalosporin (ceftriaxone®, Sandoz, Egypt) was injected for 7 successive days at a dose of 25 mg/kg b.w. *i.m*. The owners were advised to instill a tobramycin/dexamethasone combination (Tobradex®, Alcon, Egypt) for 7 days.

### Lens-induced uveitis (LIU) without ocular hypertension

Lens-induced anterior uveitis was diagnosed in 25 eyes and the treatment protocol included bulbar subconjunctival injection of 0.4 ml of dexamethasone sodium phosphate (Dexamethasone® 8 mg/2ml, Amriya pharmaceuticals, Egypt) at 3 days interval for 9 days (4 injections). The treatment also included topical administration of tropicamide1% (mydriacyl®, Alcon, Egypt) and tobramycin/dexamethasone combination (Tobradex®, Alcon, Egypt) 4–6 times a day for 3 weeks.

### Lens-induced uveitis with secondary glaucoma

Lens-induced uveitis (LIU) with secondary glaucoma (uveitic glaucoma) was diagnosed in 15 eyes and the treatment protocol included 4 systemic injections of dexamethasone sodium phosphate (0.1 mg/kg b.w. i.v.) at 3 days intervals. Instillations of tobramycin/dexamethasone combination (Tobradex®; Alcon, Egypt), timolol maleate 0.5% (timolol®; EIPICO, Egypt), and tropicamide1% (mydriacyl®, Alcon, Egypt) were performed to each eye 4 times a day for 2 successive weeks.

### Lens capsule rupture

Five eyes with traumatic scleral rupture were diagnosed in this study. These cases were associated with anterior capsule rupture and anterior luxation of the lens. All eyes were presented with acute injury and anterior lens luxation. The treatment included intracapsular lens extraction and closure of the scleral defects with 8 − 0 polyglactin (coated vicryl®; Ethicon, USA) in a simple interrupted pattern.

## Results

### Animals

The 60 dogs (90 eyes) enrolled in the present study included 12 Griffons (20%), 10 Siberian Huskies (16.7%), 8 Cocker Spaniels (13.3%), 7 Pekingese dogs (11.7%), 5 Mongrels (8.3%), 4 each of Golden Retrievers, Great Danes and German Shepherds (6.7%, each), and 2 each of Rottweilers, Jack Russell Terriers and Yorkshire Terriers (3.3%, each). Thirty-two were males and 28 were females. All the dogs were sexually intact, except for 15 females that were spayed. The mean (± SD) age of the dogs at initial evaluation was 3.65 (± 2.4) years (range, 1–12 years). Thirty dogs (50%) were admitted with bilateral eye diseases. The other 30 dogs admitted with unilateral eye affections included 18 (30%) with right-sided and 12 (20%) with left-sided eye diseases.

### Ophthalmic findings

Collectively, lens luxation and subluxation were diagnosed in 45 eyes (25 with anterior lens luxation, 15 with subluxation, and 5 with posterior lens luxation). Lens-induced uveitis without glaucoma was diagnosed in 25 eyes. Lens-induced uveitis with secondary glaucoma and lens capsule disruption were diagnosed in 15 and 5 eyes, respectively.

#### Lens luxation/subluxation with or without associated glaucoma

**Fig. 1:**
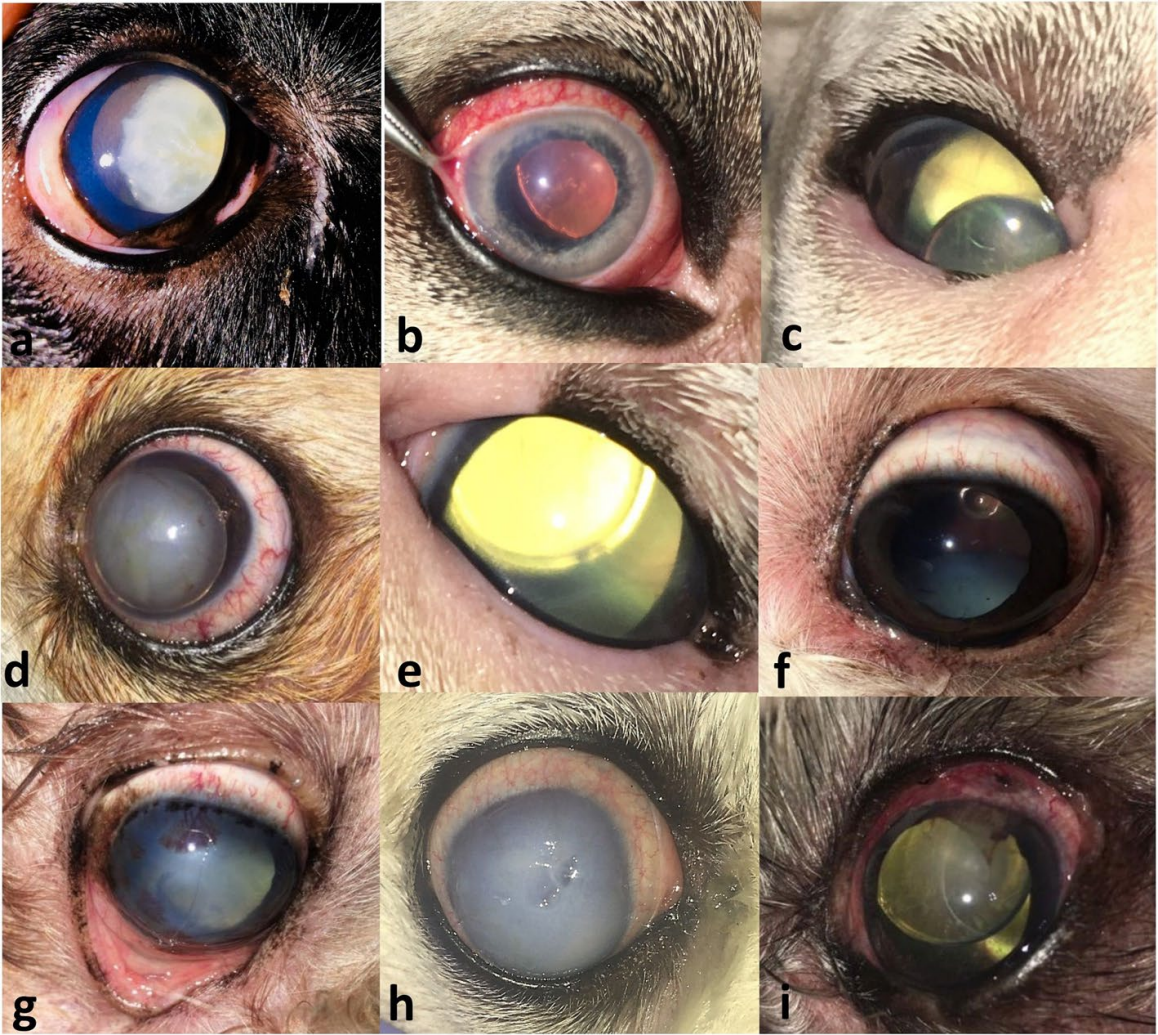
Clinical presentation of lens subluxation and luxation without or with secondary glaucoma. Lens subluxation without (**a**) and with (**b**) glaucoma. Anteriorly luxated lenses without (**c**) and with (**d**) glaucoma. Posteriorly luxated lenses with glaucoma (**e**, **f**, and **g**) and corneal involvement (**g**). Diffuse corneal oedema associated with lens luxation (**h**). Primary glaucoma with secondary lens luxation (i, note the characteristic distal aphakic crescent)

Lens luxation and subluxation likely developed as a result of cataracts, senile nuclear sclerosis, zonular degeneration, and/or trauma. Congenital lens luxation was not identified in this study. Lens luxation and subluxation with accompanying glaucoma were diagnosed in 35/45 eyes (77.8%). The mean (± SD) values of intraocular pressure at initial presentation were 41.8 (± 6.1) mmHg (range, 35–60 mmHg), 44.1 (± 5.9) mmHg (range, 38–50 mmHg), and 52.0 (± 7.4) mmHg (range, 40–60 mmHg) in cases admitted with anteriorly luxated, subluxated, and posteriorly luxated lenses, respectively. All dogs with lens luxation and subluxation had a history of long-term cataracts. Ten out of 45 eyes (22.2%) had a history of trauma and secondary lens luxation. The mean (± SD) time after trauma was 3.4 (± 2.3) days (range, 1–8 days). All luxated and subluxated lenses (45 eyes) lost their transparency, and the transparency of 15 corresponding corneas (33.3%) was not affected. The remaining 30 eyes (66.7%) showed corneal oedema and vascularisation due to endothelial damage. Clinical presentation of lens luxation and subluxation with or without glaucoma is demonstrated in Fig. 1. The distribution of dogs by breed, sex, age, affected eyes, lens localization, complications, and treatments is summarized in Table 1.

**Table 1:** Distribution of patients (33 dogs, 45 eyes) with lens luxation and subluxation with or without glaucoma according to signalment, affected eye(s), lens localization, and treatment

Breed (No)	No. of affected eyes	Sex	Age (y)	Affected eye(s)	Lens localization	Glaucoma	Treatment
**Griffon (6)**	8	M	6	Both	Subluxation	With	Miotic + PGA
		M	7	Both	Anterior luxation	With	ICLE
		M	9	OS	Anterior luxation	With	ICLE
		F	7	OS	Posterior luxation	With	Miotic + PGA
		F	8	OS	Subluxation	Without	Miotic
		F	5	OD	Anterior luxation	Without	ICLE
**Siberian Husky (4)**	5	M	1	Both	Subluxation	With	Miotic + PGA
		M	2	OS	Subluxation	With	Miotic + PGA
		F	2.5	OS	Anterior luxation	With	ICLE
		F	3	OD	Subluxation	Without	Miotic
**Cocker Spaniel (6)**	8	M	2	OD	Posterior luxation	With	Miotic + PGA
		M	2	OS	Anterior luxation	Without	ICLE
		M	1	Both	Subluxation	With	Miotic + PGA
		M	3	Both	Subluxation	With	Miotic + PGA
		M	2	OS	Anterior luxation	With	ICLE
		M	4	OD	Subluxation	Without	Miotic
**Pekingese (3)**	3	F	3	OD	Anterior luxation	With	ICLE
		M	4	OD	Posterior luxation	With	Miotic + PGA
		F	2	OD	Anterior luxation	With	ICLE
**Mongrel (3)**	4	M	1	OS	Subluxation	Without	Miotic
		M	1.5	OD	Posterior luxation	With	Miotic + PGA
		M	5	Both	Anterior luxation	With	ICLE
**Golden retriever (2)**	2	M	2	OD	Anterior luxation	Without	ICLE
		F	2	OD	Anterior luxation	Without	
**Great Dane (4)**	5	F	5	OD	Posterior luxation	With	Miotic + PGA
		F	6	Both	Subluxation	With	Miotic + PGA
		M	2	OS	Anterior luxation	With	ICLE
		F	1	OD	Anterior luxation	With	ICLE
**German Shepherd (1)**	2	M	3	Both	Anterior luxation	With	ICLE
**Rottweiler (1)**	2	M	2	Both	Anterior luxation	Without	ICLE
**Jack Russell (1)**	2	F	6	Both	Anterior luxation	With	ICLE
**Yorkshire terriers (2)**	4	F	3	Both	Anterior luxation	With	ICLE
		M	5	Both	Anterior luxation	With	ICLE

*M *Male, *F *Female, *OD *Oculus dexter (right eye), *OS *Oculus sinister (left eye), *PGA *Prostaglandin analog, *ICLE *Intracapsular lens extraction

#### Lens-induced uveitis (LIU)

**Fig. 2:**
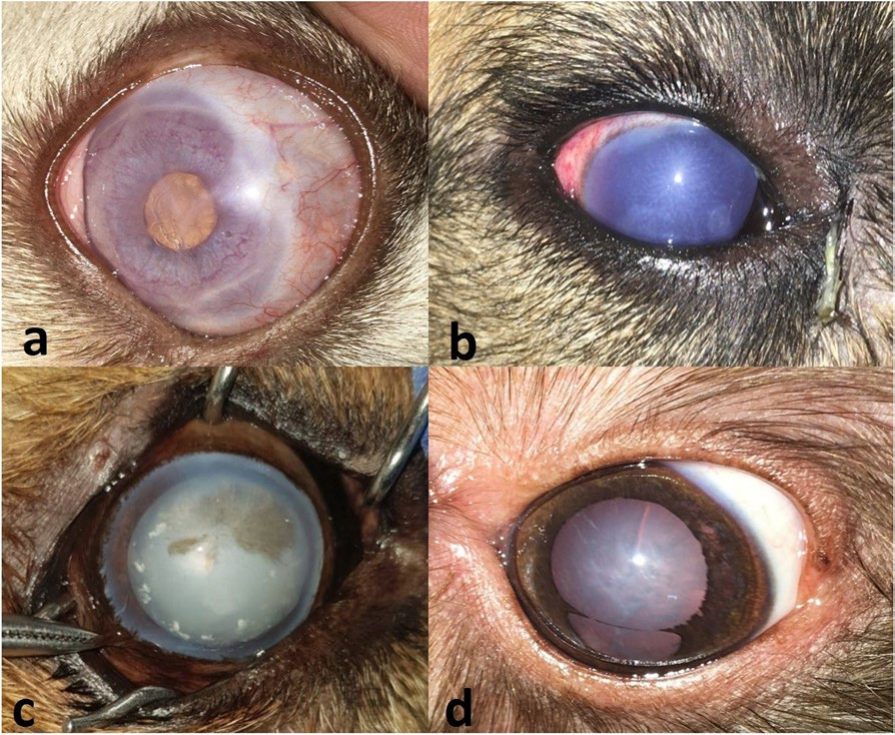
Clinical presentation of lens-induced uveitis illustrating miosis and cataract (**a**), diffuse corneal oedema (**b**), lipid flare (**c**), and chronic iris atrophy and mature cataract (**d**)

A total of 40 eyes (23 dogs) were diagnosed with LIU. Fifteen out of the 40 eyes (37.5%) had associated lens-induced uveitis with secondary glaucoma. Lens-induced uveitis without evidence of glaucoma was diagnosed in 25/40 eyes (62.5%), 20 eyes (80%) had acute uveitis and 5 eyes (20%) showed chronic iris atrophy. Ocular pain manifested by blepharospasm and photophobia was the most commonly recorded sign. Among the 25 eyes that had no evidence of glaucoma, corneal oedema was observed in 15 eyes (60%), and lipid flare (due to elevated triglycerides/cholesterol) was identified in 6 eyes (24%). Moreover, 4 eyes (16%) with LIU showed no evidence of any associated complications. The distribution of dogs with LIU with or without secondary glaucoma is demonstrated in Table 2. The clinical presentation of lens-induced uveitis is shown in Fig. 2.

**Table 2:** Distribution of patients (23 dogs, 40 eyes) with lens-induced uveitis according to signalment, affected eye(s), and associated complications

Breed (No)	No. of affected eyes	Sex	Age (y)	Affected eye(s)	Complications
**Griffon (4)**	8	M	8	Both	Corneal oedema
		M	9	Both	Corneal oedema
		M	10	Both	Uveitic glaucoma
		M	12	Both	Corneal oedema
**Siberian Huskie (6)**	9	M	1	Both	Corneal oedema
		M	1.5	Both	Corneal oedema
		M	1.5	Both	Corneal oedema
		F	3.5	OD	-
		F	2	OD	Uveitic glaucoma
		F	1.5	OS	-
**Cocker Spaniel (2)**	4	F	3	Both	Corneal oedema
		F	2	Both	Lipid flare
**Pekingese (4)**	8	M	3	Both	Lipid flare
		M	4	Both	Lipid flare
		M	2	Both	Uveitic glaucoma
		F	1.5	Both	Uveitic glaucoma
**Mongrel (1)**	2	F	2	Both	Uveitic glaucoma
**Golden retriever (2)**	4	F	4	Both	-
		F	4	Both	Uveitic glaucoma
**German Shepherd (2)**	3	M	4	OD	Corneal oedema
		F	2	Both	Uveitic glaucoma
**Rottweiler (1)**	1	F	3	OD	Uveitic glaucoma
**Jack Russell (1)**	1	M	5	OS	Uveitic glaucoma

*M *Male, *F *Female, *OD *Oculus dexter (right eye), *OS *Oculus sinister (left eye)

#### Lens-induced uveitis with secondary glaucoma

**Fig. 3:**
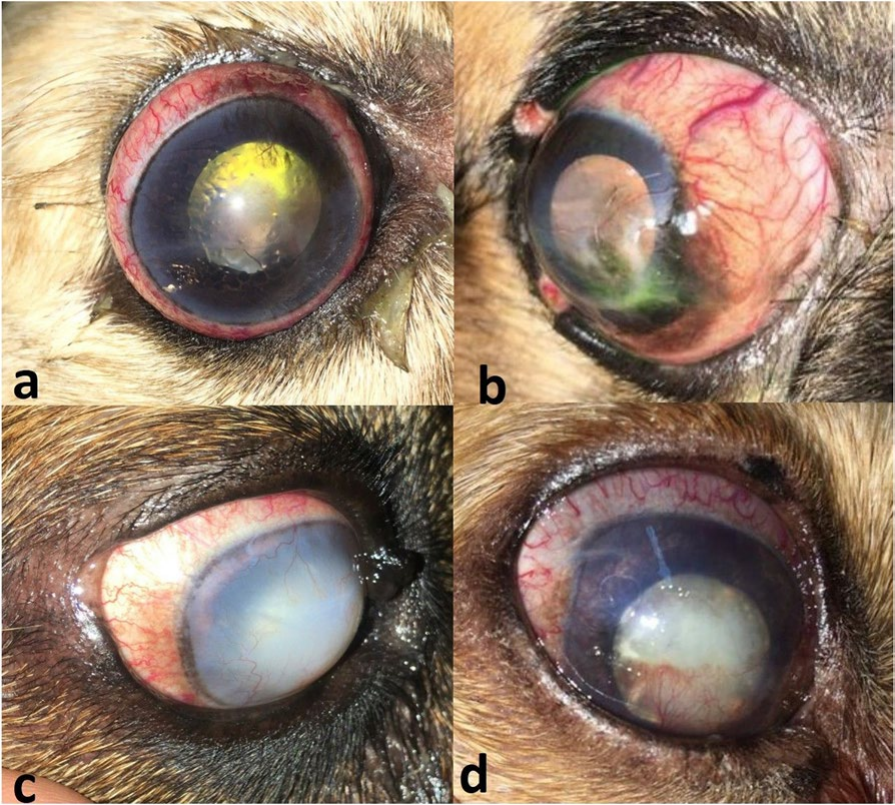
Clinical presentation of uveitic glaucoma illustrating buphthalmos, scleral injection, and chronic glaucoma secondary to resorbed cataract (**a** & **b**), secondary glaucoma due to the anteriorly luxated cataractous lens and iris atrophy (**c**), and intumescent cataract and chronic pupillary block glaucoma (**d**)

Lens-induced uveitis (LIU) with secondary glaucoma was diagnosed in 15 cases with a long-standing cataract. Ten out of 15 eyes were presented with hyper mature cataracts whereas the remaining 5 eyes were presented with morganian cataracts. The mean (± SD) IOP at the initial presentation was 35.9 (± 3.17) mmHg (range, 31–41 mmHg). The most common presenting signs included pain (evidenced by blepharospasm and lacrimation), corneal oedema, corneal vascularisation or ulceration, and congestion of the conjunctival or episcleral blood vessels (Fig. 3).

#### Anterior capsule rupture

**Fig. 4:**
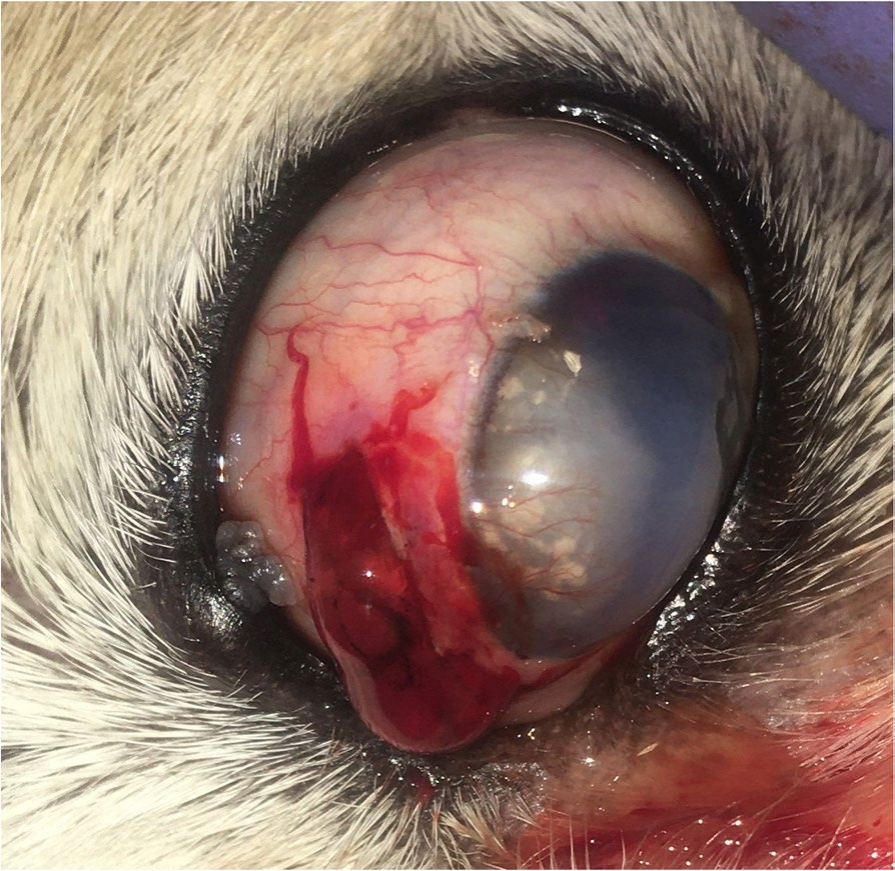
Scleral rupture with a secondary dislocated cataractous lens

Four dogs (5 eyes) were presented with acute traumatic scleral injury and subsequent rupture of the corresponding anterior lens capsule and anterior lens luxation (Fig. 4). Furthermore, confrontation with a cat before clinical signs has most likely resulted in perforating trauma to the globe by the cat’s claw.

### Ultrasonographic and laboratory findings

**Fig. 5:**
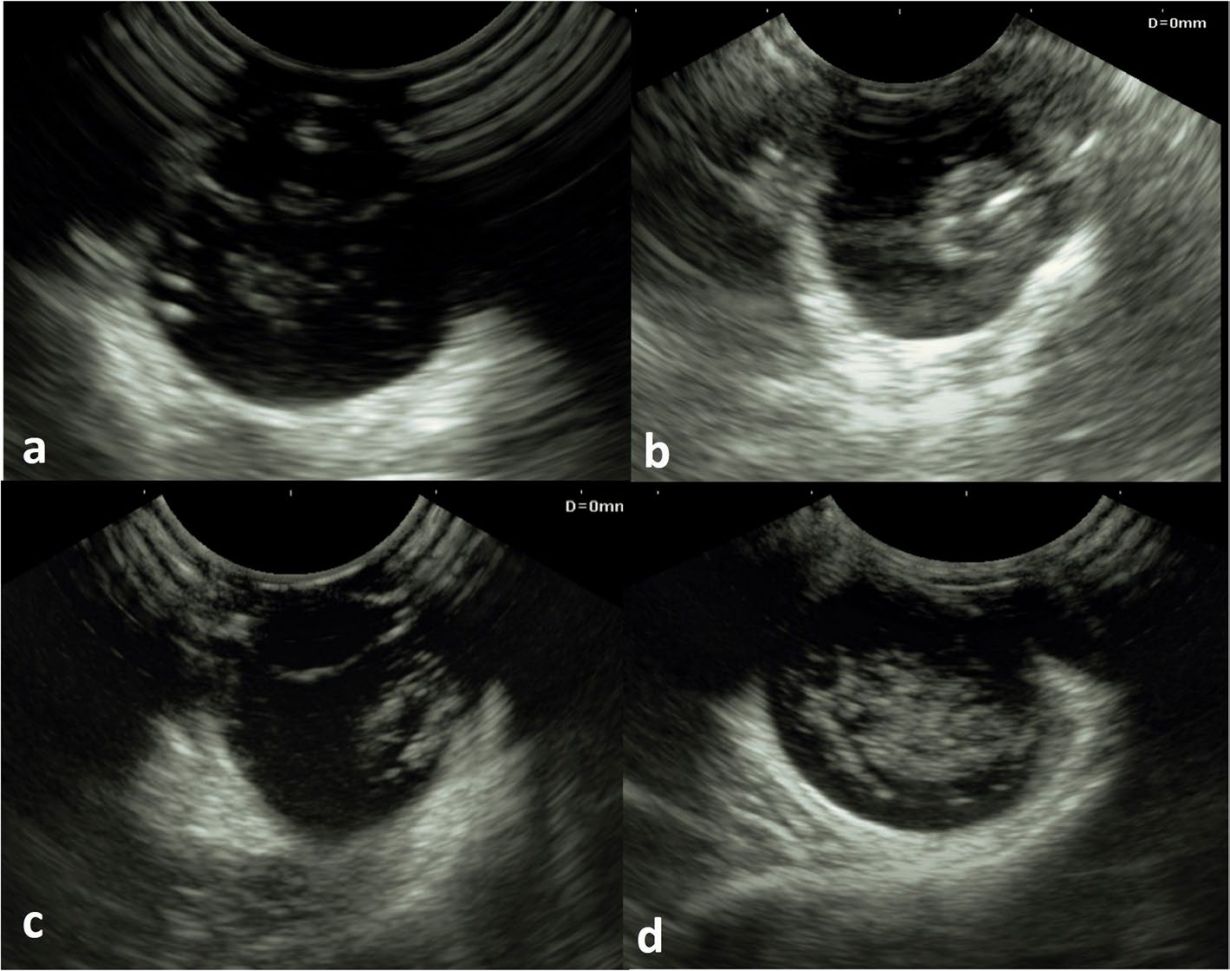
Ultrasonographic images of lens-related ocular emergencies. **a** Lens-induced uveitis and secondary phaco anaphylactic endophthalmitis showing hyperechoic cataractous lens, thick hypoechoic iris, thin hyperechoic lens capsule, and hypoechoic opacities in the vitreous. **b** & **c** Posteriorly luxated cataractous lenses with secondary glaucoma. (d) Posterior segment complications with lens luxation showing amorphous echogenicity (indicating vitreal hemorrhage and organization), anechoic retrovitreal space (indicating vitreal detachment), and hypoechoic subretinal exudate (indicating retinal detachment)

Lens dislocation, phaco anaphylactic endophthalmitis, glaucoma, vitreal haemorrahge and detachments, and retinal detachment were the recorded lens-related ocular abnormalities identified on ultrasonography (Fig. 5). The position of the dislocated lens was precisely determined via ultrasonography. Vitreal detachment was diagnosed through the presence of anechoic retrovitreal space due to the organization of vitreal haemorrahge. Vitreal haemorrahge appeared as amorphous echogenic particles within the vitreous which were mobile on real-time examination. Retinal detachment was identified through the existence of echogenic punctate or curvilinear material within the vitreous chamber and/or hypoechoic subretinal exudate. The reported cholesterol/triglycerides values for the three cases were (360/520, 400/430, and 340/460 mg/dl).

### Treatment outcomes

**Fig. 6:**
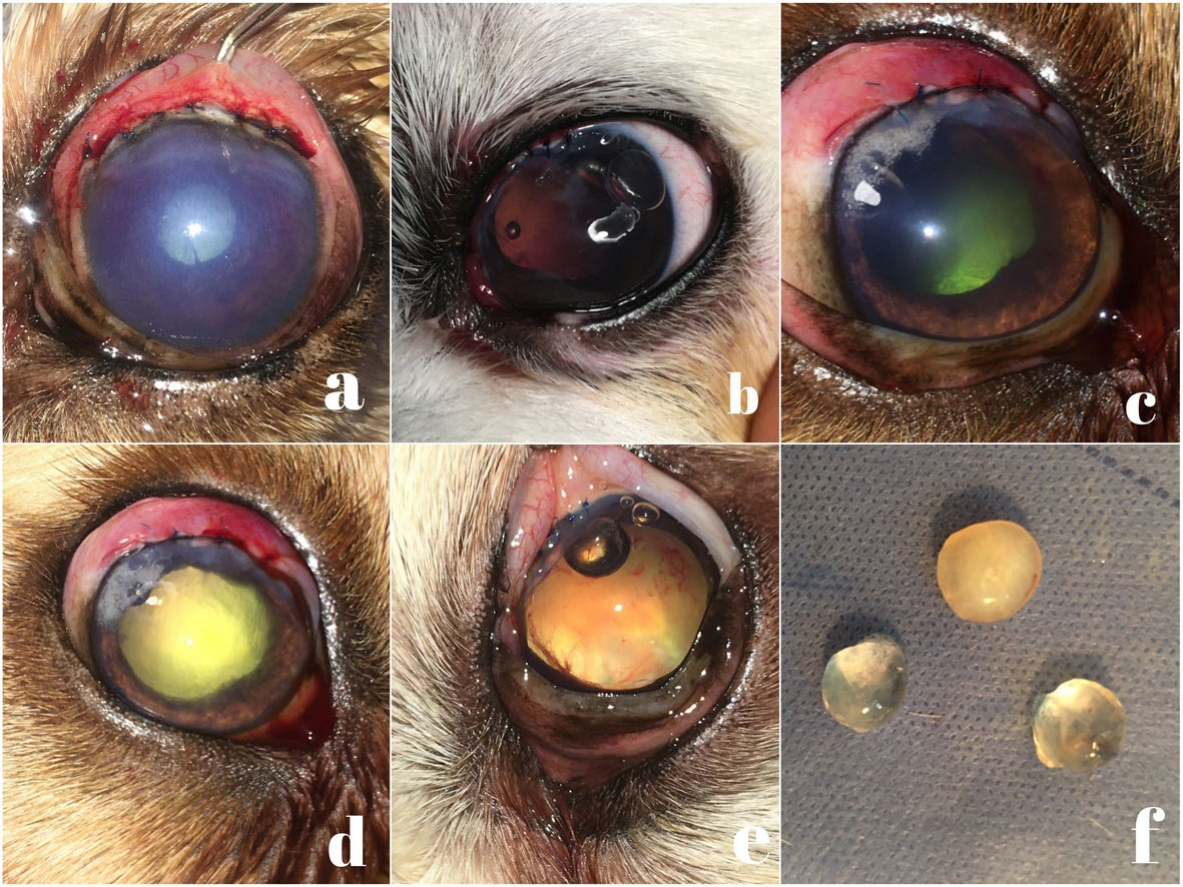
Immediate postoperative images after successful removal of the anteriorly luxated lenses (**a**, **b**, **c**, and **d**). Note the difference in transparency between lenses. Non-cataractous lenses have lost part of their transparency whereas, old cataractous lenses showed a complete lack of transparency

**Fig. 7:**
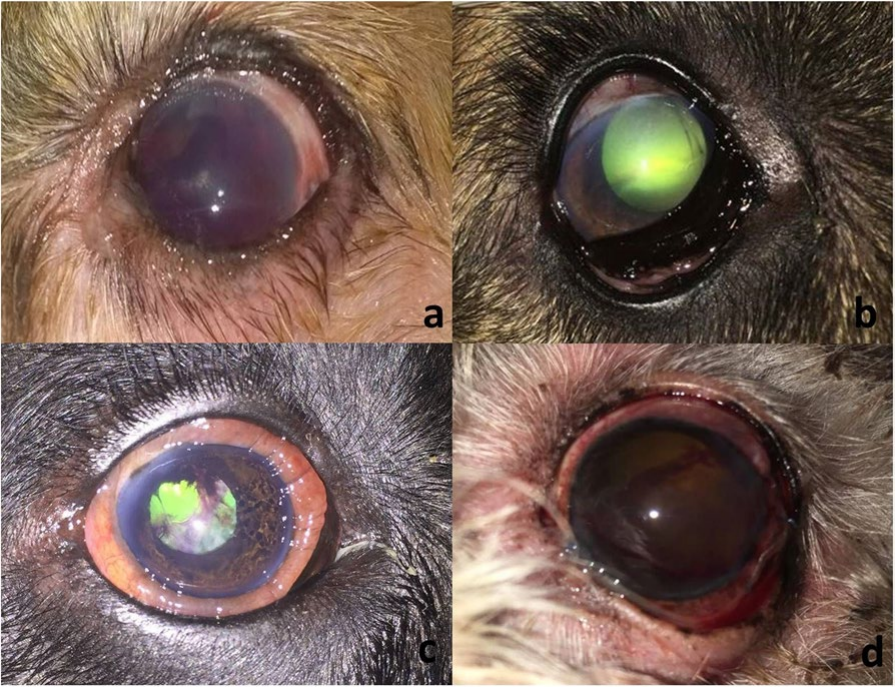
Short-term follow-up images after removal of anteriorly luxated lenses showing persistent corneal oedema (**a**), tapetal hyper-reflectivity (**b**), and remnants of the anterior lens capsule (**c**), 3 months postoperatively, and vitreal hemorrhage with secondary glaucoma and buphthalmos (**d**), one month postoperatively

Regarding eyes with subluxated lens and secondary glaucoma (11 eyes), the mean (± SD) IOP reduced to 21.5 (± 2.8) mmHg (range, 17–26 mmHg), and all eyes retained the potential for visual. However, a weekly follow-up exam revealed progressive anterior lens luxation in 5 out of 11 eyes (45.5%), and subsequent intracapsular lens extraction was performed. The 4 eyes diagnosed with lens subluxation without glaucoma showed persistence of good visual potential and no evidence of progressive lens subluxation. As for patients with posterior lens luxation and secondary glaucoma (5 eyes), the mean (± SD) IOP markedly decreased to 33.8 (± 3.4) mmHg (range, 28–38 mmHg). These patients were initially presented with vision impairment, most likely due to retinal atrophy or detachment, vitreal hemorrhage, vitreous syneresis, or corneal oedema. The anteriorly luxated lenses were successfully removed from the corresponding 25 eyes (Fig. 6). The mean (± SD) IOP of the 19 eyes diagnosed with secondary glaucoma reduced to 18.2 (± 2.1) mmHg (range, 15–22 mmHg) one week postoperatively. Hyphema and corneal oedema were recorded in 18/25 eyes (72%) immediately after lens extraction and were completely resolved by the end of the 3rd week of surgery in only 6/18 eyes (33.3%). The visual outcome was poor in 18/25 eyes (72%) with persistent corneal oedema, glaucoma, vitreal hemorrhage, vitreous displacement, and retinal detachment (Fig. 7). In 7/25 eyes (28%), the vision and quality of life were improved; however, the short-term follow-up conducted every month revealed tapetal hyper-reflectivity in 4 dogs, an abnormally positioned pupil in 2 dogs, and an existence of lens capsule remnants across the pupil in one dog.

As for the 15 eyes diagnosed with lens-induced uveitis with secondary glaucoma, the mean IOP reduced to 22.4 (± 3.7) mmHg in 8 eyes (53.3%) after one week of follow-up, with resolution of the associated symptoms and acceptable vision improvement. The remaining 7 eyes (46.7%) were however unresponsive to treatment with marked intraocular inflammation. These seven cases were treated surgically by exenteration. The clinical symptoms associated with lens-induced uveitis were resolved in 15 out of 25 eyes (60%) at the end of the 3rd week of treatment, and the vision was completely preserved in these eyes. The remaining 10 eyes including the three dogs with elevated serum triglycerides and cholesterol levels have developed secondary uncontrollable glaucoma and were treated surgically by exenteration. All dogs presented with anterior capsule rupture in the current study had lost their vision and developed phthisis bulbi (atrophied non-functional eye).

## Discussion

Ocular emergencies are common presenting complaints in veterinary and human ophthalmology. However, most ophthalmic emergencies can be treated and stabilized until a specialist ophthalmologist can be consulted. Most ocular emergencies involve loss of vision, compromised globe integrity, or severe ocular pain. This study demonstrates a wide variety of lens-related ocular emergencies in dogs as well as the short-term outcomes after treatment of these cases. The reported ocular emergencies were lens luxation or subluxation, lens-induced uveitis, and anterior capsule rupture. Glaucoma has previously been reported as a secondary complication to lens luxation or instability and lens-induced uveitis [19]. Lens luxation can be classified into congenital, or secondary luxation [20]. The primary causes of secondary lens luxation include glaucoma, uveitis, trauma, zonular degeneration, hyper-mature cataract, and intraocular tumors [20]. In viewing the results of the present study, lens luxation secondary to hyper-mature cataract or senile changes was diagnosed in 35 out of 45 eyes (77.8%). Moreover, trauma was also reported to be a cause of lens luxation as 10 eyes (22.2%) were presented with lens luxation after lateral head trauma. Congenital lens luxation was not identified in the present study based on the age of onset, breed predilection, and the absence of micro-phakia.

Dislocation of the crystalline lens, as an ocular emergency, plays a dominant role in developing different types of glaucoma and consequent loss of vision [21]. The relationship between lens luxation and secondary glaucoma was documented in 1945 by Formston who studied over 100 dogs (90 Wire Fox Terriers and 10 Sealyhams) [22]. Primary lens luxation, as occurs in Terriers and certain other breeds, may result in a pupillary block with acute elevation in IOP [19]. Primary glaucoma tends to occur in young, middle-aged to older dogs of certain breeds, and lens subluxation or luxation does not occur until the globe becomes buphthalmic and the lens zonules stretch beyond the breaking point (secondary luxation). Similarly, lens luxation and glaucoma can result from the development of mature cataracts in many dog breeds [19]. In viewing the results of the present study, the mean (± SD) age of dogs at initial evaluation was 3.65 ± 2.4 years. Secondary glaucoma due to a dislocated lens and primary glaucoma with secondary lens luxation were diagnosed in 77.8% (35/45) of our enrolled eyes. The rise in IOP secondary to lens luxation could be attributed to blockage of the pupil by the anteriorly displaced swollen lens as well as blockage of the trabecular meshwork by lens proteins [21]. The characteristic aphakic crescent identified in the present study suggests the existence of broken lens zonules secondary to primary glaucoma [19]. Moreover, all dislocated lenses in this study were cataractous.

Corneal oedema secondary to lens luxation was reported in 30 cases (66.7%) and was attributed to endothelial damage or impaired function of the corneal endothelium from an elevated IOP [19]. Lens-induced uveitis, as an ocular emergency, was diagnosed in 40 eyes in this study with 15 eyes (37.5%) developing secondary glaucoma (uveitic glaucoma). The cause of glaucoma was most likely due to a phaco-anaphylactic response to lens material [13]. This is in agreement with previous reports which suggested that cataract formation and associated lens-induced uveitis are frequent causes of secondary glaucoma in dogs [7]. Applanation tonometry has been reported to be important in monitoring lens-induced uveitis and possible secondary glaucoma in cataractous dogs that for some reason did not have cataract surgery [23]. Moreover, the presence of lipid flare in diabetic patients as a result of elevated triglycerides or cholesterol, or both, may be the first indicator of inflammation [8]. The common sequelae after full-thickness corneoscleral injury in dogs included traumatic cataract, anterior lens capsule rupture, secondary glaucoma, and septic endophthalmitis [13]. Anterior lens luxation from a ruptured capsule is considered an ocular emergency that may develop even after the healing of such sequelae. In our study, all dogs with anterior capsule rupture had lost their vision and their eyes progressed to phthisis bulbi (atrophied non-functional eye).

B-mode ultrasonography is a safe, non-invasive rapid, and reliable method of evaluating the interior of the eye globe. It has proven its efficacy in determining the position of the dislocated lens and posterior segment abnormalities, particularly in cases with diffuse corneal oedema [24]. In the current study, the recorded lens-related ocular abnormalities involved lens dislocation, phaco anaphylactic endophthalmitis, glaucoma, vitreal haemorrahge or detachment, and retinal detachment. The cause of retinal detachment was determined through ultrasonographic evidence of subretinal exudate and vitreoretinal traction. Vitreal haemorrahge appeared as amorphous, multifocal to coalescing echogenic spots within the vitreous which were mobile on the real-time exam [25]. Vitreal detachment was most likely the result of the organization of vitreal haemorrahge and was evidenced by the presence of anechoic retrovitreal space [25].

Surgical interventions were performed in 15/45 eyes (33.3%) with lens subluxation and in 5/45 eyes (11.1%) with posterior lens luxation. However, 5 out of 11 eyes (45.5%) with lens subluxation and secondary glaucoma progressed to anterior lens luxation and the surgery was then performed as recommended in a previous report [19]. Nevertheless, these 5 eyes had lost their vision, thus medical management along with periodical assessment could be crucial for these patients to possibly achieve a better outcome. The IOP was controlled in 6/11 eyes (54.5%) and these eyes retained the potential for visual. The reduction in the IOP, following the use of Latanoprost, was attributed to the increased uveoscleral flow [26]. All eyes with posteriorly luxated lenses had lost their vision as a result of retinal atrophy or detachment, vitreal haemorrahge, vitreous syneresis, or persistent corneal oedema. Glover et al., (1995) reported high success rates at 4–6 weeks after intra-capsular lens removal via cryo-extraction through a clear 160-degree corneal incision [20]. Nonetheless, this technique was declined one year postoperatively by the same study [20]. In the present study, 72% of the eyes with anterior lens luxation had lost their vision even after successful lens extraction. The causes of vision loss were possibly attributed to secondary glaucoma, vitreous hemorrhage or displacement, retinal detachment, and persistent corneal oedema. Lens-induced uveitis (LIU) is a common ocular emergency that may result in loss of vision due to secondary uveitic glaucoma. In our study, the vision was completely lost in 7/15 eyes (46.7%) with uveitic glaucoma and 10/25 eyes (40%) with unresponsive LIU. The potential limitation of the current study was that most enrolled patients were admitted during the late stages of the disease. Additional limitations were the limited number of enrolled cases and short-term follow-up. Therefore, future investigation on a larger population and longer follow-up is still warranted to overcome these limitations.

## Conclusion

Crystalline lens pathology can cause a wide variety of ocular emergencies that may result in blindness. Ocular emergencies that were found to be associated with vision impairment included lens subluxation, anterior or posterior lens luxation, uveitic glaucoma, unresponsive LIU, and traumatic rupture of the anterior lens capsule. Early diagnosis and appropriate treatment are the most important factors for handling lens-related emergencies in dogs that generally develop due to negligence.

## Data Availability

The data sets supporting our results are included in the article. Row data are available in the Department of Small Animal Surgery and Ophthalmology, Faculty of Veterinary Medicine, Cairo University, Egypt upon official request to the corresponding author (aymostafa@cu.edu.eg).

## Abbreviations

LROE: Lens-related ocular emergencies
OD: Oculus dexter
OS: Oculus sinister
ICLE: Intracapsular lens extraction
IOP: Intraocular pressure
LIU: Lens-induced uveitis
PGA: Prostaglandin analog
